# T44. A RANDOMISED, DOUBLE-BLIND, PLACEBO-CONTROLLED TRIAL OF THE EFFECTS OF VITAMIN B12, B6 AND FOLIC ACID ON COGNITION AND SYMPTOMS IN FIRST-EPISODE PSYCHOSIS: THE VITAMINS IN PSYCHOSIS STUDY

**DOI:** 10.1093/schbul/sby016.320

**Published:** 2018-04-01

**Authors:** Kelly Allott, Patrick McGorry, Hok Pan Yuen, Colin O’Donnell

**Affiliations:** 1 Orygen, The National Centre of Excellence in Youth Mental Health; 2 Donegal Mental Health Service

## Abstract

**Background:**

Vitamin B12, vitamin B6 and folic acid are homocysteine-reducing agents. People with schizophrenia have been found to have increased homocysteine levels. Elevated homocysteine has been associated with impaired cognition. Previous research in chronic schizophrenia has shown that supplementation with folate plus vitamin B12 can improve cognition and clinical symptoms. Whether homocysteine lowering agents are effective in first-episode psychosis is unknown. The aim of this study was to investigate if adjunctive vitamin B12, B6 and folic acid can lower homocysteine and improve symptomatology and cognition in people with first-episode psychosis.

**Methods:**

This was a randomised, double-blind, placebo-controlled trial conducted at the Early Psychosis Prevention and Intervention Centre (EPPIC) in Melbourne, Australia. One hundred and twenty patients aged 15–26 years with first-episode psychosis consented and were randomised to receive folic acid 5mg, vitamin B12 0.4mg, and vitamin B6 50mg or placebo, each taken once-daily for 12 weeks as an adjunct to antipsychotic medication. Co-primary measures were change in cognition as measured by a composite score from a battery of 11 tests and total symptomatology (PANSS) over 12 weeks. Secondary outcomes included additional cognitive, symptom, functioning, tolerability and safety measures.

**Results:**

Of the 120 participants randomised in the study, 20 dropped out with no follow-up assessments and were excluded from analysis. Of the remaining 100 participants, 52 were in the vitamins group and 48 the placebo group. At baseline, the two treatment groups had lower levels of folate and vitamin B12 intake than healthy controls, but did not differ from each other. Vitamin B12, B6 and folic acid reduced homocysteine levels in the vitamin group over 12 weeks. The homocysteine lowering effects of the vitamins did not confer a major advantage over placebo therapy in improving the co-primary PANSS (p=.75) or composite cognition (p=0.79) outcomes over 12 weeks. There was a significant difference between groups among females in the cognitive domain of speed of processing (p=.049) and attention/vigilance (p=0.002), in which the mean performance of the placebo group declined over 12 weeks, whereas performance in the vitamin group remained showed improvement.

**Discussion:**

Folic acid, B12 and B6 supplementation appears well tolerated and safe in first-episode psychosis and lowers homocysteine levels in this population. However, supplementation may not offer extra benefits to all patients with first-episode psychosis, with the possible exception of speed of processing and attention/vigilance. Although previous research suggests that males preferentially benefit, our findings suggest that there may be a specific beneficial effect on cognition for females with first-episode psychosis.

